# Measuring trends in age at first sex and age at marriage in Manicaland, Zimbabwe

**DOI:** 10.1136/sti.2008.033431

**Published:** 2009-03-13

**Authors:** I Cremin, P Mushati, T Hallett, Z Mupambireyi, C Nyamukapa, G P Garnett, S Gregson

**Affiliations:** 1 Department of Infectious Disease Epidemiology, Imperial College London, UK; 2 Biomedical Research and Training Institute, Harare, Zimbabwe

## Abstract

**Objective:**

To identify reporting biases and to determine the influence of inconsistent reporting on observed trends in the timing of age at first sex and age at marriage.

**Methods:**

Longitudinal data from three rounds of a population-based cohort in eastern Zimbabwe were analysed. Reports of age at first sex and age at marriage from 6837 individuals attending multiple rounds were classified according to consistency. Survival analysis was used to identify trends in the timing of first sex and marriage.

**Results:**

In this population, women initiate sex and enter marriage at younger ages than men but spend much less time between first sex and marriage. Among those surveyed between 1998 and 2005, median ages at first sex and first marriage were 18.5 years and 21.4 years for men and 18.2 years and 18.5 years, respectively, for women aged 15–54 years. High levels of reports of both age at first sex and age at marriage among those attending multiple surveys were found to be unreliable. Excluding reports identified as unreliable from these analyses did not alter the observed trends in either age at first sex or age at marriage. Tracing birth cohorts as they aged revealed reporting biases, particularly among the youngest cohorts. Comparisons by birth cohorts, which span a period of >40 years, indicate that median age at first sex has remained constant over time for women but has declined gradually for men.

**Conclusions:**

Although many reports of age at first sex and age at marriage were found to be unreliable, inclusion of such reports did not result in artificial generation or suppression of trends.

In the era of the HIV pandemic, the focus of analyses regarding timing of first sex and marriage has broadened from investigating their role as proximate determinants of fertility to encompass their associations with risk of acquiring HIV.^1^^–^3 Identifying trends in the timing of age at first sex and marriage is essential for evaluating the impact of the investment made in HIV prevention interventions to promote abstinence (by delaying first sex), and to assess the contribution of these changes to any downturn in the epidemic.^4^^–^7 Delays in reported age at sexual debut have coincided with declines in the prevalence of HIV in Uganda and Zimbabwe.^8^^–^12 Mathematical modelling, however, has indicated that these changes are unlikely to have contributed substantially to reductions in lifetime risk of infection or HIV incidence.^13^

The relationship between age at marriage and risk of infection has received attention. Recently, Bongaarts argued that late marriage can facilitate the spread of HIV via a long period of premarital sexual activity.^14^ However, it is also possible for early marriage to pose an increased risk of infection if marriage leads to regular unprotected sex in serodiscordant partnerships.^15^

In general, the age at which first sex occurs lies within the later teenage years.^16^ ^17^ Thus, changes in the median age at first sex of just a year or two are considered epidemiologically significant. However, even modest degrees of social desirability bias, recall bias, digit preference or random misreporting could potentially generate spurious trends. For example, social desirability would be expected to bias reports of age at first sex upwards. Slaymaker and Buckner found that declines in reporting of sex before age 15 in Zambia were partially due to a change in reporting bias, when comparing two survey methodologies and accounting for the sociodemographic composition of the survey samples.^18^ The Manicaland cohort study in Zimbabwe, which asked individuals about the timing of first sex and marriage in three survey rounds spanning a period of 8 years, presents an opportunity to monitor trends in age at first sex and marriage as well as to gauge the consistency of reporting which may inform on the accuracy and reliability of these data.

The objectives of this paper are to (1) quantify the level of inconsistent reporting and its influence on observed trends; (2) identify reporting biases; (3) describe the overall trends in age at first sex and age at marriage; and (4) describe the length of time spent single (between first sex and marriage) in this population.

## METHODS

### Study design

The Manicaland HIV/STD Prevention Project is a population-based open cohort study in the Manicaland province of eastern Zimbabwe. The study design and procedures have been outlined in detail previously.^12^ Briefly, participants were recruited from 12 predominantly rural study sites which consisted of 4 subsistence farming areas, 2 small towns, 2 roadside trading areas and 4 forestry, coffee and tea estates. Local residents were enumerated initially in a household census. Eligible individuals (those present in a local household for at least four nights in the past month and at the same time one year ago) were then invited to participate in the study. This baseline survey took place in a phased manner from July 1998 to February 2000. This survey was repeated as a first follow-up from 2001 to 2003 and second follow-up from 2003 to 2005.

Men aged 17–54 years and women aged 15–44 years were recruited with only one member of each cohabiting marital union selected at random to participate. At the second follow-up survey, extended entry criteria allowed men and women aged 15–54 years and both members of a marital union to participate. In the second survey (from site five onwards) and the third survey, migrants into the study areas were invited to participate.

### Behaviour data

Participants were interviewed using a structured questionnaire on sociodemographic characteristics and sexual behaviour. Interviews were led by social science graduates who were familiar with the local language and culture but were not known locally. Interviewers were trained to adopt an informal and non-prejudicial approach and to build up a good rapport with respondents. Interviewers were matched for sex with respondents. An informal confidential voting interview (ICVI) method was used to collect sensitive behavioural data as a means of reducing social desirability bias.^19^ Participants placed slips containing their responses into a “voting” box, thus ensuring confidentiality. All data on age at first sex and age at first marriage were collected using the face-to-face interview method, even when the ICVI method was used to collect subsequent behavioural data in the same interview.

Participants were asked if they had been married or were in a long-term or cohabiting relationship, which we classify together as being “married”. In the first round of the survey, relationships of ⩾6 months were defined as long-term, but in the subsequent two rounds this was changed to be relationships of ⩾12 months. Ever married individuals were asked their age when they first entered such a relationship. In the section on sexual behaviour, participants were asked initially how old they were when they had sex for the first time. Subsequent questions on sexual behaviour were asked in order of increasing sensitivity.

### Analysis of data

A number of methods have been developed to analyse data on age at first sex (which also apply to age at marriage). The data used are current status (the current age of the individual and whether they have ever had sex) and recall (retrospective reports of age at first sex).^20^ Survival analyses combine the two sources. These techniques can also be adapted to analyse data from successive surveys to assess trends in age at first sex and distinguish underlying trends from reporting errors.^21^

Survival analysis techniques were employed to analyse timing of first sex, with individuals represented as surviving until first sex. In this scheme the failure event is starting sex and follow-up time is censored if individuals had not started sex by the time of their last interview. Censoring is independent (of the probability that sex occurs), since no information on sexual behaviour was obtained prior to recruitment. Kaplan-Meier failure curves were plotted to illustrate the cumulative onset of sexual activity. This procedure was also followed when analysing trends in age at marriage.

Age at first sex and age at marriage were reported as years in integers, which correspond to age at last birthday at those events. A random proportion of a year was added to each age to capture the distribution of exact ages within a year. Reports of age at first sex and age at marriage were classified according to consistency to assess the quality of reporting. Four categories were defined to classify reports: (1) consistent; (2) inconsistent: can be corrected; (3) inconsistent: can be estimated; and (4) unreliable: cannot be estimated or corrected (table 1).

**Table 1:** Definitions of reliable and unreliable reporting

Category	Number of age-at-event reports			Imputed response	
	1	2	3		
Consistent	Report ⩽ age at survey	Both reports same AND both reports ⩽ age at survey	All reports same AND all reports ⩽ age at survey	Reported age	Reliable
Inconsistent: can be corrected	N/A	Reports differ by 1 year AND both reports ⩽ age at survey	2 ages reported which differ by 1 year AND both reports ⩽ age at survey	Older of the 2 ages	
Inconsistent: can be estimated	N/A	1 report ⩽ age at survey	⩾50% of reports same AND these reports ⩽ age at survey	Most frequent report that is ⩽ age at survey	
Unreliable: cannot be estimated or corrected	Report >age at survey	Reports differ by >1 year AND both reports ⩽ age at survey OR both reports > age at survey	<50% of reports same OR ⩾50% reports same AND reports > age at survey	Mean of all reported ages	Unreliable

A number of non-mutually exclusive classifications were observed for those individuals with three reports (827/3270)—that is, when two reports were consistent and the other report differed by 1 year. In such cases the consistently reported age was used. These criteria were also applied when classifying reports of age at marriage. A detailed description of these classifications, by frequency of reporting, is given by Wringe *et al*.^22^

All analyses were carried out using Stata Version 10 (Stata Corp, College Station, Texas, USA).

## RESULTS

The numbers of participants at the first, second and third surveys were 9530, 8210 and 16 266, respectively. In total, 23 517 individuals were interviewed in at least one survey, of which 16 680 were interviewed in only one survey, 3185 were interviewed in two surveys and 3652 were interviewed in all three. Most individuals who were surveyed only once were new entrants at the third survey round in which the eligibility criteria for the study were expanded, as described above.

### Age at first sex

All respondents were asked what age they were when they first had sex. Of all the surveyed individuals, 23.2% (5446/23 517) reported never having had sex. Slightly more men than women reported never having had sex (24.3% vs 22.3%, χ^2^ test, p<0.001). Of the individuals who provided a report of age at first sex, 69.3% (12 448/17 953) only reported in one round. A small proportion of individuals did not respond to the question regarding age at first sex (63 (0.66%) in round 1, 36 (0.44%) in round 2 and 48 (0.30%) in round 3).

**Figure 1:**
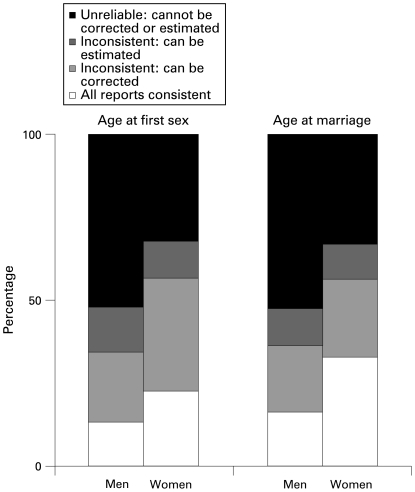
Consistency of reporting age at first sex and age at marriage among men and women aged 15–54 years who reported in at least two surveys.

Reports were classed as unreliable for 32.1% of women and 52.2% of men who reported age at first sex in at least two surveys (fig 1). Of these unreliable reports, 31.2%, 21.2% and 15.9% of reports differed by 2, 3 and 4 years, respectively. The remaining 31.6% of reports differed by >4 years. Similar levels of unreliable reporting were observed among only those who participated in all three surveys. The quality of reporting among those in all three surveys was very similar across birth cohorts, as described by Wringe *et al*.^22^ Excluding unreliable reports had little effect on the failure curves compared with analysing all reports (regardless of consistency) for both men and women. Median age at first sex remained largely unchanged when unreliable reports were excluded (table 2).

**Table 2:** Median and IQR for age at first sex and age at marriage by reliability of data and birth cohort for men and women

Birth cohort	Age at first sex		Age at first marriage	
	All data	Reliable data	All data	Reliable data
	Median (IQR)	Median (IQR)	Median (IQR)	Median (IQR)
Men				
1950–9	20.1 (18.3–22.4)	19.9 (18.0–22.2)	24.5 (22.0–27.0)	24.6 (21.9–27.0)
1960–9	19.6 (17.9–21.6)	19.5 (17.8–21.7)	24.7 (22.0–27.4)	25.0 (22.0–27.7)
1970–9	19.0 (17.3–20.9)	19.0 (17.2–20.9)	23.2 (21.2–25.2)	23.4 (21.3–25.4)
Women				
1950–9	19.0 (17.3–20.6)	18.9 (17.2–20.5)	19.1 (17.4–20.8)	18.9 (17.2–20.7)
1960–9	18.7 (17.0–20.6)	18.7 (17.0–20.6)	18.9 (17.2–20.9)	18.8 (17.0–20.8)
1970–9	19.0 (17.4–20.8)	19.0 (17.5–20.8)	19.3 (17.7–21.1)	19.3 (17.7–21.2)

IQR, interquartile range.

**Figure 2:**
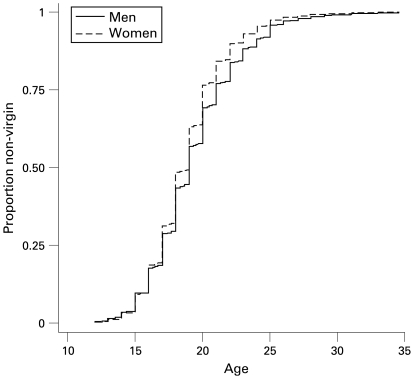
Kaplan-Meier failure curve for age at first sex for men and women. Data include all reports after corrections and estimations for inconsistent reports.

Age at first sex was significantly higher for men than for women (log-rank test for equality of survivorship functions: χ^2^ = 110, p<0.001; fig 2). Over all the surveys, 12.6% of men and 12.0% of women reported experiencing first sex at 15 years or younger.

**Figure 3:**
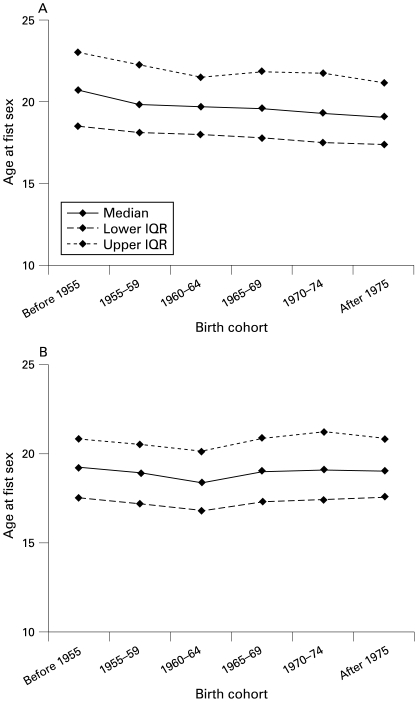
Median estimates of reported age at first sex by 5-year birth cohort for (A) men and (B) women. IQR, interquartile range. Data include all reports after corrections and estimations for inconsistent reports. The upper value of the IQR could not be calculated for those born after 1975 as 60% of men and 60% of women born after 1975 reported not having had sex.

Plotting medians by birth cohort indicated that age at first sex declined gradually among men, from 20.7 years for those born before 1955 to 19.1 years for those born after 1975 (fig 3A). For women the median age at first sex remained relatively constant between 19.2 and 19.0 years (fig 3B).

The reports from those reporting in at least two surveys were then combined and analysed in a life table format to compare reports by the same individuals as they age. The median age at first sex should remain unchanged as a cohort ages. Among those in older birth cohorts (1965–9 to before 1955), trends in age at first sex remained unchanged as these individuals aged (reading across the table), indicating the absence of reporting biases (table 3). However, among those in younger birth cohorts (1970–4 and after 1975), reported age at first sex increased as they aged.

**Table 3:** Median age at first sex and number reporting (N) for men and women reporting in at least two surveys by 5-year birth cohort and age group

Birth cohort	Men							Women						
	15–19	20–24	25–29	30–34	35–39	40–44	45–49	15–19	20–24	25–29	30–34	35–39	40–44	45–49
Before 1955						20.0	20.0						19.3	19.0
						(*22*)	(224)						(*42*)	(436)
1955–9					19.5	20.0	20.0					19.0	19.0	18.7
					(*23*)	(257)	(172)					(*50*)	(667)	(544)
1960–4				19.0	19.0	19.0					18.3	18.0	18.0	
				(*48*)	(379)	(239)					(*65*)	(816)	(671)	
1965–9			19.3	19.0	19.3					18.0	18.3	18.0		
			(*45*)	(352)	(231)					(*69*)	(656)	(496)		
1970–4		18.8	19.0	19.0					18.0	18.3	18.3			
		(*68*)	(609)	(388)					(*62*)	(750)	(599)			
After 1975	17.0	18.0	18.3					17.0	18.0	18.6				
	(415)	(1047)	(458)					(448)	(1103)	(586)				

Data include all reports after corrections and estimations for inconsistent reports have been made. Small sample sizes are indicated in italic, along the leftmost diagonal for men and for women. These are low due to the timing of the surveys in relation to birth cohorts (eg, the majority of individuals in the 1970–4 birth cohort had aged past the 20–24 age group when they were surveyed).

### Age at marriage

Across all respondents, 36.9% (8676/23 517) reported never having been married. A higher proportion of men than women had never been married (50.3% vs 26.8%, χ^2^ test, p<0.001). Participants who reported having ever been married or in a long-term relationship were asked at what age they first entered such a relationship. Of these, 43 (0.72%), 45 (0.85%) and 59 (0.55%) did not respond to the question regarding age at marriage in the first, second and third surveys, respectively. Of the individuals who reported an age at marriage, 68% (9874/14 508) only reported it in one round.

Reports were classed as unreliable for 33.2% of women and 52.5% of men who reported age at marriage in at least two surveys (fig 1). Of these unreliable reports, 26.5%, 17.8% and 13.7% of reports differed by 2, 3 and 4 years, respectively. The remaining 41.9% of reports differed by >4 years. Excluding unreliable reports when plotting failure curves did not change the overall trends when compared with trends based on all reports (regardless of consistency). The median age at marriage remained largely unchanged when unreliable reports were excluded (table 2).

**Figure 4:**
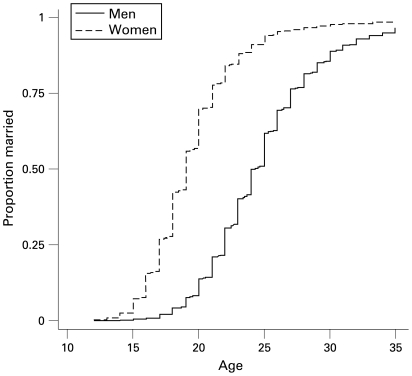
Kaplan-Meier failure curve for age at marriage for men and women. Data include all reports after corrections and estimations for inconsistent reports.

**Figure 5:**
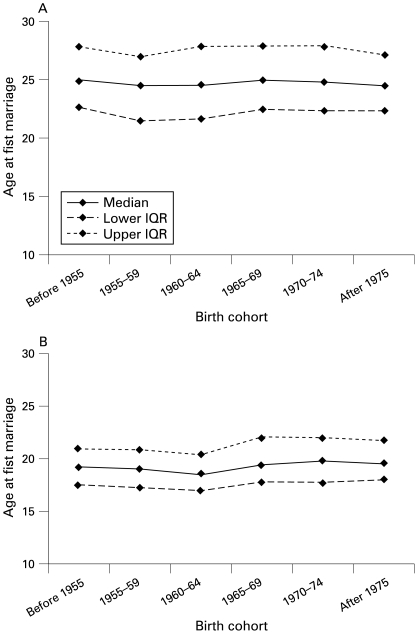
Median estimates of reported age at marriage by 5-year birth cohort for (A) men and (B) women. IQR, interquartile range. Data include all reports after corrections and estimations for inconsistent reports. The IQR and median age at marriage could not be calculated for men born after 1975 as only 20% of men in this birth cohort are (or had been) married. The upper value of the IQR could not be calculated for women born after 1975 as 52% of women in this birth cohort are (or had been) married.

Men marry at significantly older ages than women (log-rank test χ^2^ = 5647, p<0.001; fig 4). A stable age at marriage among men and women is also illustrated by plotting the median age for each birth cohort (fig 5).

The reports from those reporting in at least two surveys were then combined and analysed in a life table format. Men generally reported an increased age at marriage as they aged, particularly those in the youngest birth cohort (reading across the table; table 4). Women in older birth cohorts (1955–9 and before 1955) reported decreasing ages at marriage as they aged. However, for women in younger birth cohorts (1965–9 to after 1975), the reported age at marriage increased as they aged.

**Table 4:** Median age at marriage and number reporting (N) for men and women reporting in at least two surveys by 5-year birth cohort and age group

Birth cohort	Men							Women						
	15–19	20–24	25–29	30–34	35–39	40–44	45–49	15–19	20–24	25–29	30–34	35–39	40–44	45–49
Before 1955						23.8	24.0						19.0	18.7
						(*22*)	(219)						(*41*)	(425)
1955–9					21.0	23.0	23.0					19.0	18.0	18.0
					(*22*)	(254)	(168)					(*49*)	(661)	(541)
1960–4				23.5	23.0	23.0					18.0	18.0	18.0	
				(*45*)	(364)	(234)					(*63*)	(803)	(664)	
1965–9			24.2	23.7	23.3					18.0	18.7	18.3		
			(*42*)	(324)	(216)					(*67*)	(645)	(492)		
1970–4		22.6	23.0	23.0					18.0	19.0	18.7			
		(*50*)	(441)	(307)					(*59*)	(710)	(573)			
After 1975	19.5	21.0	22.0					17.0	18.0	18.3				
	(*55*)	(313)	(244)					(357)	(963)	(542)				

Data include all reports after corrections and estimations for inconsistent reports. Small sample sizes are indicated in italic, along the leftmost diagonal for men and for women. These are low due to the timing of the surveys in relation to birth cohorts (eg, the majority of individuals in the 1970–4 birth cohort had aged past the 20–24 age group when they were surveyed).

### Time spent single after sexual debut

**Figure 6:**
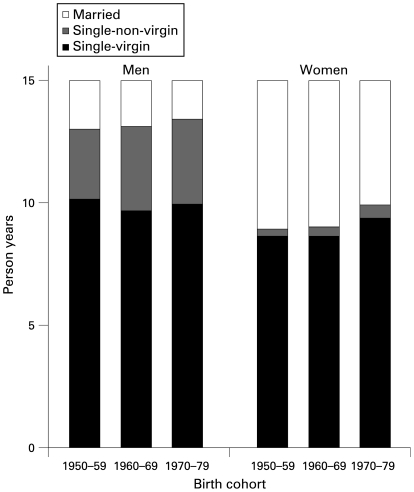
Distribution of person-years spent as a virgin, sexually active and unmarried and married between the ages of 10 and 25 years for men and women.

The average number of person-years spent between first sex and marriage has remained constant for men and women (fig 6). The mean (SD) duration of time spent single after sexual debut is much longer for men than for women (4.1 (4.1) years vs 0.3 (1.6) years). A greater proportion of women than men indicated that their first sexual experience was within marriage; 69.8% (6748/9666) and 15.3% (729/4760) of married women and men, respectively, reported age at first sex to coincide with age at marriage.

## DISCUSSION

Including reports of age at first sex and age at marriage identified as unreliable in survival analyses did not alter our results, suggesting that much of the unreliability may be attributable to random misreporting rather than systematic biases such as social desirability bias. This absence of alteration of trends indicates that the observed trends can be interpreted with some confidence, despite the high levels of misreporting. Furthermore, this provides some reassurance when interpreting trends in serial cross-sectional data, such as from Demographic and Health Surveys (DHS) where such inconsistencies cannot be identified. However, consistency of reporting does not always signify accuracy; an individual may consistently report an incorrect age at first sex, a bias which cannot be detected.

In this rural Zimbabwean population, women initiate sex and enter marriage at younger ages than men but spend much less time between first sex and marriage. Comparisons across all surveys by birth cohorts which span a period of >40 years indicate that the median age at first sex has remained constant over time among women but has declined gradually among men. Median age at marriage has remained relatively stable for both men and women. However, tracing birth cohorts between surveys revealed reporting biases. It is possible that some individuals (particularly young people) deliberately report an older age at first sex due to exposure to HIV prevention campaigns promoting delaying age at first sex and abstinence, in addition to the pressure of other social norms. With respect to reported age at first sex, one may expect decreasing social desirability bias and increasing recall bias as an individual ages. Such effects may generate the similar levels of unreliable reporting observed across all birth cohorts.

We previously reported delays in sexual debut among teenage men and women in the first 3 years of follow-up.^12^ In this earlier analysis we compared trends in the percentage of individuals reporting having had sex within a small population subgroup who were starting sex at that time (17–19-year-old men and 15–17-year-old women). These age restrictions were chosen to analyse an entirely new group of maturing adolescents at each survey, thus focusing on very recent trends. These previous findings, using current status data only, are therefore not directly comparable to the longer-term trends in median age at first sex in the entire population presented here by birth cohorts spanning a period of >40 years.

**Take-home messages**

- High levels of reports of age at first sex and age at marriage were identified as unreliable among men and women attending multiple surveys.
- Excluding unreliable reports did not alter the observed trends in age at first sex and age at marriage.
- Comparing birth cohorts over a 40-year time span, median age at first sex has remained constant over time among women but has declined gradually among men.
- Median age at marriage has remained relatively stable for both men and women.

In Zimbabwe, information on timing of sexual debut has been collected in four DHS surveys since 1988, and median age at first sex has been calculated by province for the most recent three surveys.^23^^–^26 In Manicaland, median ages among women aged 25–49 years are relatively constant at 18.3, 18.2 and 18.4 years in 1994, 1999 and 2005–6, respectively. Among men aged 25–54 years (25–49 years in the 2005–6 survey) the median age at first sex increased from 19.5 years in 1994 to 22.0 years in 1999 and subsequently decreased to 20.6 years in 2005–6. Thus, the medians calculated for women in this study population are a good representation of those in the wider population in the Manicaland province whereas, for men, the medians calculated for this study population are generally younger. Our estimates of median age at first sex are relatively high in comparison with other African countries such as Kenya, Tanzania, Uganda and Zambia where median age at first sex ranges from 16.4 to 18.1 years.^21^

The substantial gender differential in age at marriage observed here is not surprising as women are expected to marry younger than men in Shona society.^27^ Women may seek to marry at a relatively young age in order to obtain the security associated with marriage and subsequent motherhood. Men are expected to obtain employment in order to afford bridewealth, thus re-enforcing the expectation that they wait until reaching their mid 20s before marrying. Recall bias may influence quality of reporting of age at marriage, particularly among older individuals. In this society, marriage is a long process and individuals may be unable to distinguish their age when the actual ceremony took place from that when bridewealth was paid, from when the relationship begun. The biases identified in reporting of age at marriage may also be partially attributable to the change in the definition of a long-term relationship.

A mathematical modelling study parameterised for this population has shown that the effect of delaying age at first sex on subsequent risk of HIV infection depends on the length of time spent single (between first sex and marriage).^13^ When sexually active and unmarried, the risk of infection is double that when married (2% and 1% per person-year at risk, respectively).^13^ Thus, the extended duration of time spent sexually active and single among men, and the brief duration of time spent sexually active and single among women observed here, support previous findings in this population that, in general, young men acquire HIV from premarital relations (as well as extramarital relations) whereas, for women (who typically spend much less time single), the strongest determinants of infection are their spouse’s behaviours.^28^ ^29^

Previous cross-sectional analyses (using the first round of data) investigating age at first sex in relation to subsequent risk of HIV infection found that, among both men and women in this population, riskier sexual behaviour was observed among those reporting an early age at first sex (younger than 18 years) than in those who reported an older age of sexual debut.^3^ However, for men, an early age at first sex which coincided with age at marriage did not lead to subsequent riskier behaviour compared with men who initiated sex at an older age. This suggests that, among men, early marriage may be somewhat protective against the riskier effects of early age at first sex. However, the change in age at first sex observed here among men may not translate into an additional risk of infection because the average number of years spent single (between first sex and marriage) has remained the same. Thus, the circumstances in which first sex occurs may be more important than the timing.
